# Developmental Exposure to a Human-Relevant Polychlorinated Biphenyl Mixture Causes Behavioral Phenotypes That Vary by Sex and Genotype in Juvenile Mice Expressing Human Mutations That Modulate Neuronal Calcium

**DOI:** 10.3389/fnins.2021.766826

**Published:** 2021-12-06

**Authors:** Sunjay Sethi, Kimberly P. Keil Stietz, Anthony E. Valenzuela, Carolyn R. Klocke, Jill L. Silverman, Birgit Puschner, Isaac N. Pessah, Pamela J. Lein

**Affiliations:** ^1^Department of Molecular Biosciences, School of Veterinary Medicine, University of California, Davis, Davis, CA, United States; ^2^Department of Psychiatry and Behavioral Sciences, School of Medicine, University of California, Davis, Davis, CA, United States; ^3^The MIND Institute, University of California, Davis, Davis, CA, United States

**Keywords:** *FMR1* permutation, gene-environment interaction, neurodevelopmental disorders, ryanodine receptor, sex differences, social behavior, T4826I mutation, ultrasonic vocalization

## Abstract

Polychlorinated biphenyls (PCBs) are putative environmental risks for neurodevelopmental disorders. Here, we tested two hypotheses: (1) developmental exposure to a human-relevant PCB mixture causes behavioral phenotypes relevant to neurodevelopmental disorders; and (2) expression of human mutations that dysregulate neuronal Ca^2+^ homeostasis influence sensitivity to behavioral effects of developmental PCB exposures. To test these hypotheses, we used mice that expressed a gain-of-function mutation (T4826I) in ryanodine receptor 1 (*RYR1*), the X-linked fragile X mental retardation 1 (*FMR1*) CGG repeat expansion or both mutations (double mutant; DM). Transgenic mice and wildtype (WT) mice were exposed to the MARBLES PCB mix at 0, 0.1, 1, and 6 mg/kg/day in the maternal diet throughout gestation and lactation. The MARBLES PCB mix simulates the relative proportions of the 12 most abundant PCB congeners found in the serum of pregnant women at increased risk for having a child with a neurodevelopmental disorder. We assessed ultrasonic vocalizations at postnatal day 7 (P7), spontaneous repetitive behaviors at P25-P30, and sociability at P27-P32. Developmental PCB exposure reduced ultrasonic vocalizations in WT litters in all dose groups, but had no effect on ultrasonic vocalizations in transgenic litters. Developmental PCB exposure significantly increased self-grooming and decreased sociability in WT males in the 0.1 mg/kg dose group, but had no effect on WT females in any dose group. Genotype alone influenced ultrasonic vocalizations, self-grooming and to a lesser extent sociability. Genotype alone also influenced effects of PCBs on sociability. PCB levels in the brain tissue of pups increased in a dose-dependent manner, but within any dose group did not differ between genotypes. In summary, developmental PCB exposure phenocopied social behavior phenotypes observed in mice expressing human mutations that modify intracellular Ca^2+^ dynamics, and expression of these mutations alleviated PCB effects on ultrasonic vocalizations and repetitive behavior, and modified the dose-response relationships and sex-dependent effects of PCB effects on social behavior. These findings suggest that: (1) developmental PCB exposure causes behavioral phenotypes that vary by sex and genotype; and (2) sex-specific responses to environmental factors may contribute to sex biases in the prevalence and/or severity of neurodevelopmental disorders.

## Introduction

The prevalence of neurodevelopmental disorders, and in particular, autism spectrum disorder, is increasing worldwide (Landrigan et al., 2012; Baio et al., 2018), and several studies have documented that this is not due solely to increased awareness and expanded diagnostic criteria (Bishop et al., 2008; Coo et al., 2008; Hertz-Picciotto and Delwiche, 2009). The rapid rise in the prevalence of autism spectrum disorder coupled with evidence that single genetic factors alone account for only a percentage of cases (El-Fishawy and State, 2010; Herbert, 2010; Hallmayer et al., 2011; Weintraub, 2011; Landrigan et al., 2012) strongly support a role for environmental factors in determining individual risk and/or severity of these and other neurodevelopmental disorders (Sealey et al., 2016; Lyall et al., 2017). Given the social and economic burden of autism spectrum disorder and, more broadly, neurodevelopmental disorders, on individuals, families, and society (Barrett et al., 2012; Lavelle et al., 2014), there is significant interest in identifying factors that modify individual risk for neurodevelopmental disorders. In contrast to genetic risks, which are currently challenging to reverse, environmental factors are modifiable risk factors; therefore, identifying specific environmental factors that confer risk for neurodevelopmental disorders may provide rational approaches for mitigating the severity or preventing clinical outcomes.

Polychlorinated biphenyls (PCBs) are a class of persistent organic pollutants that are widely recognized as neurodevelopmental toxicants (Schantz et al., 2003; Boucher et al., 2009; Berghuis et al., 2015; Pessah et al., 2019). More recently, PCBs have been identified as environmental risk factors for neurodevelopmental disorders (reviewed in Pessah et al., 2019; Xi and Wu, 2021). Although PCB production was banned worldwide in the early 2000s, human exposures to these pollutants continue, in part because of the environmental persistence of the legacy PCBs found in the commercial PCB mixture, but also because of their release from PCB-containing hazardous waste sites, aging equipment, and building materials (Hornbuckle and Robertson, 2010; Klosterhaus et al., 2014; Grimm et al., 2015). Humans are also exposed to contemporary PCBs produced as inadvertent byproducts of contemporary pigment and dye production (Hu and Hornbuckle, 2010; Guo et al., 2014; Herkert et al., 2018). The predominant routes of human exposure to both legacy and contemporary PCBs are ingestion and inhalation (Ampleman et al., 2015), and women of childbearing age continue to have quantifiable PCB levels in their serum (Koh et al., 2015; Granillo et al., 2019).

One mechanism posited to explain how environmental factors interact with genes of susceptibility to increase risk for neurodevelopmental disorders is the convergence of environmental and genetic factors on signaling pathways that regulate phenotypes relevant to neurodevelopmental disorders (Lein, 2015). We previously demonstrated that a subset of non-dioxin-like PCB congeners that sensitize the ryanodine receptor (RyR) activate Ca^2+^-dependent signaling pathways that normally mediate activity-induced dendritic growth and synapse formation, to increase dendritic arborization and synaptic density in primary rat hippocampal and cortical neurons (Yang et al., 2009, 2014; Wayman et al., 2012a; Lesiak et al., 2014). Interestingly, increased dendritic arborization and synapse density are common in many neurodevelopmental disorders (Engle, 2010; Penzes et al., 2011; Supekar et al., 2013), and a large number of risk genes for neurodevelopmental disorders regulate or are regulated by Ca^2+^-dependent signaling and/or regulation of dendritogenesis and synaptogenesis (Krey and Dolmetsch, 2007; Stamou et al., 2013; Grove et al., 2019). Thus, the goal of this study was to test the hypotheses that (1) developmental exposure to a human-relevant PCB mixture causes behavioral phenotypes of relevance to neurodevelopmental disorders; and (2) expression of heritable mutations that alter Ca^2+^-dependent signaling modulate the behavioral effects of developmental PCB exposure.

To test these hypotheses, we leveraged several mouse lines genetically engineered to express human mutations previously shown to alter the fidelity of neuronal Ca^2+^ signals (Keil et al., 2019b). The first is the T4826I-*RYR1* mouse, which expresses a human gain-of-function mutation in the gene that encodes *RYR1*, a transmembrane ion channel that is an essential regulator of intracellular Ca^2+^ stores (Barrientos et al., 2012; Yuen et al., 2012). Approximately 15% of the human population is estimated to carry one or more *RYR1* genetic variants (Kim et al., 2013). Gain-of-function mutations in the human *RYR1* gene underlie malignant hyperthermia susceptibility, which predisposes carriers to acute, potentially lethal, hyperthermia in response to heat stress, anesthetics, and other environmental stressors (Pessah et al., 2010; Dlamini et al., 2013). RyR1 activity is necessary for BDNF-induced remodeling of dendritic spines (Adasme et al., 2011), and activity-dependent dendritic growth and synaptogenesis (Wayman et al., 2012b; Lesiak et al., 2014). The second transgenic line we tested was the CGG mouse strain, which expresses CGG repeat expansions in the 5′ non-coding region of the fragile X mental retardation gene 1 (*FMR1*) in the premutation range (55–200 CGG repeats) (Berman et al., 2014). Expansion mutations in the *FMR1* gene are the most prevalent monogenic risk factor for neurodevelopmental disorders (Krueger and Bear, 2011; Leehey and Hagerman, 2012). Estimated prevalence of the *FMR1* premutation in the human population is 1:209 in females and 1:430 in males (Tassone et al., 2012). Primary neurons derived from the CGG mouse strain were observed to have altered dendritic morphology compared to primary neurons from wildtype (WT) littermates (Chen et al., 2010), increased resting intracellular Ca^2+^ concentrations, and abnormal patterns of spontaneous Ca^2+^ oscillations (Cao et al., 2012; Robin et al., 2017), findings that have been corroborated in human iPSC-derived neurons from *FMR1* premutation patients (Liu et al., 2012). A third strain that expresses both mutations, referred to as the double mutant (DM) mouse, was developed to investigate gene dose and gene:gene interaction effects on neurodevelopmental outcomes (Keil et al., 2019b). We previously reported that juvenile male and female DM mice exhibited significantly decreased sociability relative to WT mice (Keil et al., 2019b).

In this study, we exposed these transgenic mice and WT control mice to the MARBLES PCB mix (Sethi et al., 2019) *via* the maternal diet throughout gestation and lactation. The MARBLES PCB mix was based on the PCB congener profile detected in the serum of pregnant women at increased risk of having a child with a neurodevelopmental disorder (Hertz-Picciotto et al., 2018; Sethi et al., 2019). We assessed ultrasonic vocalizations, self-grooming and social approach as a tailored behavioral battery relevant to clinical behavioral phenotypes in neurodevelopmental disorders (Silverman et al., 2010; Ey et al., 2011). The circulating thyroid hormone levels and PCB brain burden of developmentally exposed offspring were also examined.

## Materials and Methods

### Materials

Organic peanut butter (Trader Joe’s, Monrovia, CA) and organic peanut oil (Spectrum Organic Products, LLC, Melville, NY) were purchased from Trader Joe’s (Davis, CA). The individual PCB congeners (PCB 11, 28, 52, 84, 95, 101, 118, 135, 138, 149, 153, and 180) used to make the MARBLES PCB mix were synthesized and authenticated as previously described (Li et al., 2018; Sethi et al., 2019). All PCB congeners were > 99% pure (Sethi et al., 2019). All organic solvents used for gas chromatography were of HPLC grade and obtained from Fisher Scientific (Fair Lawn, NJ, United States). PCB standards (PCB 11, 28, 52, 84, 95, 101, 118, 135, 138, 149, 153, and 180) were purchased from AccuStandard Inc. (New Haven, CT, USA). The ^13^C12-labeled 2,2′,3′,4,5-pentachlorobiphenyl (^13^C12-PCB-97) was purchased from Cambridge Isotope Laboratories (Tewksbury, MA, United States). Mirex was purchased from Sigma Aldrich (St. Louis, MO, United States). Solutions were diluted with isooctane to appropriate concentrations.

### Animals

All procedures involving animals were conducted in accordance with the NIH Guide for the Care and Use of Laboratory Animals and were approved by the University of California, Davis, Animal Care and Use Committee. Protocols conformed to the ARRIVE guidelines (Kilkenny et al., 2010). Mice were derived from transgenic mouse colonies maintained at UC Davis (Keil et al., 2019b). These included mice homozygous for the human gain-of-function mutation in *RYR1* (T4826I-*RYR1*) referred to as T4826I mice, mice homozygous (female) or hemizygous (male) for the X-linked CGG repeat expansion in *FMR1* in the premutation range (170–200 repeats), referred to as CGG mice, and double mutant (DM) mice that expressed both mutations (Keil et al., 2019b). C57Bl/6J and SVJ129 WT mice were purchased from Jackson Labs (Sacramento, CA) and crossed to generate a 75% C57Bl/6J/25% SVJ129 hybrid WT line that was used to match the genetic background of the T4826I, CGG and DM animals as determined by single nucleotide polymorphism (SNP) analysis. All animals used in this study were genotyped as previously described (Keil et al., 2019b). To generate the juvenile mice used for behavioral phenotyping, homo/hemizygous matings were used as previously described (Keil et al., 2019b).

Two weeks prior to mating, nulliparous and previously unmated dams (>6 weeks of age) were singly housed and PCB dosing was initiated. Dams were placed with a genotype-matched male overnight for mating. Males and females were separated the next day and females were checked for the presence of a copulatory plug, which was considered gestational day 0. After mating, dams were housed singly prior to parturition and with their pups after parturition. At postnatal day 2 (P2), pups were culled or cross-fostered within genotype- and dose-matched litters to ensure all litters consisted of 4–8 pups. After weaning at P21, pups were group housed with same-sex littermates prior to behavioral testing, which has been reported to not alter social approach behavior (Yang et al., 2015). All mice were group-housed in clear plastic cages containing corncob bedding and maintained on a 12 h light and dark cycle at 22 ± 2°C and 40–50% humidity. Food (Diet 5058, LabDiet, Saint Louis, MO) and water were available *ad libitum*.

### Developmental Polychlorinated Biphenyl Exposures

Humans are exposed to both higher- and lower-chlorinated PCBs *via* both ingestion and inhalation (Ampleman et al., 2015). Therefore, as the MARBLES mix included both higher- and lower-chlorinated congeners, we exposed dams to PCBs *via* the diet beginning 2 weeks prior to mating and continuing throughout gestation and lactation until pups were weaned at P21, at which time PCB exposure of the pups ceased. This timeframe corresponds to human brain development throughout gestation, e.g., rodent brain development during the first 3 weeks after birth corresponds to human brain development during the third trimester (Rice and Barone, 2000; Semple et al., 2013). The MARBLES mix was dissolved in peanut oil, which was then homogeneously mixed into peanut butter using a Bullet blender as previously described (Rude et al., 2019). Vehicle control consisted of peanut oil mixed into peanut butter. Two weeks prior to meeting, dams were randomly assigned to a dose group and dosing was initiated. Dosing continued daily throughout gestation and lactation up to P21 *via* ingestion of peanut butter containing peanut oil or the MARBLES PCB mix at doses of 0.1, 1, or 6 mg/kg/day based on daily body weight. Each dam was weighed and the amount of PCB mixture was adjusted for body weight to ensure the appropriate dose was consumed, which typically occurred within 15–20 min.

### Pup Ultrasonic Vocalizations

Ultrasonic vocalizations were recorded using Avisoft UltraSoundGate microphone and Avisoft Recorder USGH software (version 4.2, Avisoft Bioacoustics, Glienicke, Germany) and spectrograms were analyzed using Avisoft SASLab Pro software (version 5.2, Avisoft Bioacoustics) as previously described (Brielmaier et al., 2012; Berg et al., 2018). Since ultrasonic vocalizations can vary within a litter (Rieger and Dougherty, 2016), all pups in the litter were analyzed using litters of 6–8 animals. Briefly, pups at P7 were removed from their home cage, placed in a separate container of corn cob bedding within a sound attenuating chamber equipped with an Avisoft UltraSoundGate microphone. Ultrasonic vocalizations were recorded for a total of 3 min. After each recording period, body mass and temperature were recorded, after which pups were returned to their home cage. The temperature of the room was maintained at 22 ± 2°C. Ultrasonic calls were manually quantified by an experienced individual without knowledge of the experimental group. The total number of calls per pups over the 3-min recording period was averaged across all pups within the litter such that each litter was assigned a single total number of ultrasonic vocalizations that was used in statistical analyses.

### Empty Cage Observations

Empty cage observations were conducted from P25-P30 as previously described (Ellegood et al., 2021; Haigh et al., 2021). Home cages were moved into the behavioral testing room (lux level 110) and allowed at least 1 h to acclimate prior to testing. One mouse was randomly chosen from each cage and placed into a separate empty plastic cage with a plastic lid, no bedding and without a top wire rack. The assay lasted 20 min, with the first 10 min considered the habituation phase, which was not scored. The last 10 min of the recording were scored by an experienced individual without knowledge of experimental group. The only behavior observed was self-grooming. The recorder sat approximately 2 m away from the testing cage and scored the cumulative amount of time spent grooming all body regions with a stopwatch. The testing cage was thoroughly cleaned with 70% ethanol to remove any scent cues and was allowed sufficient time to dry between each mouse. Once mice underwent the self-grooming test, they remained individually housed and were used 2 day later for social approach behavior.

### Social Approach

Sociability was measured at P27-P32 using a three-chambered social box constructed from white plastic with removable doors as described previously (Silverman et al., 2010; Keil et al., 2019a). Mice were moved into the behavioral testing room (lux level 110) in their home cage and allowed at least 1 h acclimation prior to testing. The sociability assay consisted of three 10-min periods for a total of 30 min. First, animals were placed in the center of the three-chambered arena for a 10-min acclimation period. Second, in the habituation phase, the doors were removed and mice were allowed free exploration of the entire arena for 10 min. If an animal failed to explore during this time, they were removed from the study. Finally, during the last 10-min session, animals were allowed to interact with either an empty, upside-down wire pencil cup (object) or an identical cup containing a novel age- and sex-matched WT mouse. The two cups were placed on opposite sides of the arena. Placement of the object and mouse was counterbalanced between the left and right sides of the area to eliminate side bias. Both the area and the cups were cleaned with 70% ethanol and allowed to dry completely between each animal to eliminate scent cues. Animals used as novel mice were habituated to sitting under the wire cup for 10 min to avoid erratic behavior during the testing phase of the assay. Behavior was recorded and analyzed using Noldus EthoVision XT (version 11.0) automated tracking and analysis software (Noldus Information Technology Inc., Leesburg, VA).

### Brain Polychlorinated Biphenyl Measurement

Following the conclusion of behavioral testing, animals that underwent behavioral testing and untested littermates were euthanized *via* CO_2_ inhalation. Brains were rapidly dissected from the skull and sagittally bisected. One hemisphere was collected for PCB analyses and quickly flash-frozen in pre-washed glass vials and stored at −80°C until analysis. A 50-mg portion of the frozen brain tissue was placed in a microtube and homogenized in 800 μL acetonitrile using a Geno/Grinder bead homogenizer (SPEX SamplePrep LLC, Metuchen, NJ, United States). After homogenization, the tubes were vortexed, sonicated, and mechanically shaken to facilitate extraction of the PCBs. Samples were then centrifuged and the supernatant collected. The remaining pellet was resuspended and extracted twice more using a 50:50 solution of acetonitrile and isopropanol; 600 μL of the solution was used for the second extraction and 400 μL for the third. During each subsequent extraction, the tubes were vortexed, sonicated, shaken, centrifuged, and the supernatant collected. The three supernatants were filtered through a Phree Phospholipid Removal Plate (Phenomenex Inc., Torrance, CA, United States) to remove proteins and phospholipids, and then combined. The combined supernatant was evaporated under nitrogen, reconstituted in 50 μL isooctane, and then centrifuged to remove any residue. The resulting supernatant was loaded into an auto-sampler vial for GC/EI-MS/MS analysis.

An eight-point calibration curve at PCB concentrations of 2.5, 5, 10, 20, 30, 40, 60, and 80 ng/g was prepared by adding PCB analytical standards (AccuStandard Inc.) to 50 mg of untreated mouse brain homogenates derived from mice that had not undergone any experimental manipulations. Quality control (QC) samples at PCB concentrations of 12.5, 25 and 50 ng/g were prepared in the same manner. Calibrators and QCs, as well as matrix blanks and reagent blanks, were processed following the same extraction method as samples. All samples, calibrators, QCs and blanks were internal standard-corrected with a ^13^C-labeled PCB 97 internal standard (Cambridge Isotope Laboratories Inc.) at a concentration of 100 ng/g.

Extracted samples were then run as previously described (Lin et al., 2013; Sethi et al., 2017) using a Bruker Scion triple quadrupole mass spectrometer equipped with a Scion 456-GC and CP-8400 auto-sampler and series split/splitless injector (Bruker Scientific LLC, Billerica, MA, United States). The GC-MS/MS data were processed using Bruker Mass Spectrometry Working Station version 8.2 (Bruker). All analytes were quantified using the eight-point calibration curve. The peak areas were used for quantification following an internal algorithm. The limit of detection (LOD) and limit of quantification (LOQ) were defined based on signal-to-noise (S/N) ratio exceeding 3 and 10, respectively. Values found below the LOD were reported as “non-detected” (ND).

### Thyroid Hormone Measurement

Following euthanasia, blood was collected *via* cardiac puncture. Blood was allowed to clot and then centrifuged at 5,200 × *g* for 10 min. The serum was collected and immediately frozen at −80°C until analysis. Serum was thawed for analysis, and 3,3′,5-triidothyronine (T3) and thyroxine (T4) were measured by ELISA (Calbiotech, El Cajon, CA) according to the manufacturer’s protocol. Samples were run in technical duplicates with the average of the duplicates used for statistical analysis.

### Statistical Analyses

Statistical analyses were performed using GraphPad Prism software (version 9.0). A one-way analysis of variance (ANOVA) or Kruskal Wallis test with *post hoc* Holm-Sidak’s multiple comparisons test or Dunn’s test, respectively, was used to assess PCB effects within each genotype, or genotype effects within vehicle groups of each sex for ultrasonic vocalizations, grooming behavior, thyroid hormone, and PCB brain burden data compared to sex-matched controls. Social approach data were assessed using an unpaired *t*-test for parametric data, unpaired *t*-test with Welch’s correction for data with unequal variance, and a Mann-Whitney *U*-test for non-parametric data. Thyroid hormone and PCB tissue concentration data were analyzed using a one-way ANOVA, one-way ANOVA with Welch’s correction or Kruskal Wallis test with Holm-Sidak’s multiple comparisons test, Dunnett’s T3 multiple comparisons test or Dunn’s test, respectively, as indicated in the figure legends.

## Results

This study is part of an overall study designed to assess the effects of developmental exposure to the MARLES PCB mixture on multiple endpoints, including the gut microbiome and intestinal physiology (Rude et al., 2019), cytokine levels in the serum and hippocampus (Matelski et al., 2020), and dendritic arborization of hippocampal and cortical neurons (Keil Stietz et al., 2021). As reported previously, developmental PCB exposure had no effect on pregnancy rates across genotypes, which averaged 88%, or the length of time from mating to parturition (Matelski et al., 2020). While dam weight at weaning was not altered by PCB exposure, there was a significant main effect of genotype, with DM dams weighing significantly more than WT dams, T4826I dams weighing significantly more than CGG dams, and CGG dams weighing significantly less than DM dams (Matelski et al., 2020). There were no effects of developmental PCB exposure or genotype on litter size or sex ratio within the litter (Supplementary Figure 1).

### Developmental Polychlorinated Biphenyl Exposure and Genotype Effects on Ultrasonic Vocalizations

**FIGURE 1:**
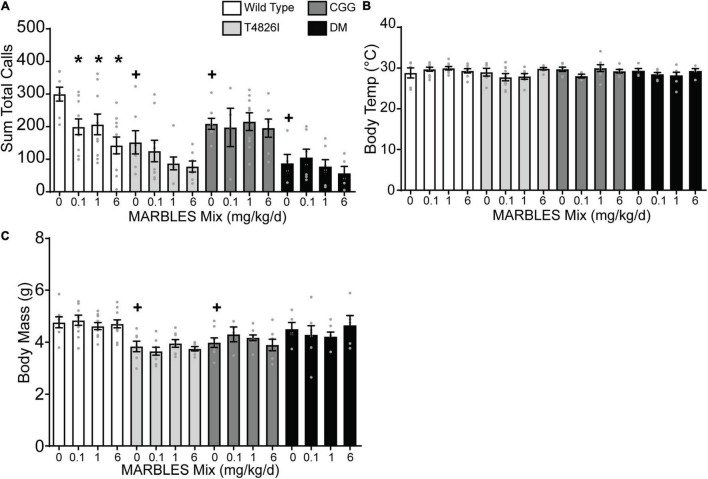
Developmental PCB exposure or expression of mutations in calcium signaling decreased the number of ultrasonic vocalizations (USVs) emitted from pups at P7. **(A)** The sum total calls made during the 3-min testing period averaged per litter. **(B)** Body temperature during the testing period averaged per litter. **(C)** Body mass during the testing period averaged per litter. Only litters of 6–8 pups were used for analysis with *n* = 4–11 litters per group. Data are presented as mean ± SEM. *Indicates significantly different from vehicle control within a genotype, + indicates significantly different from WT vehicle control at *p* < 0.05 as determined using a one-way ANOVA or Kruskal Wallis test with *post hoc* Holm-Sidak’s multiple comparisons test or Dunn’s test, respectively.

Ultrasonic vocalizations are emitted by mouse pups following separation from the dam and littermates during the first 2 weeks of life, with call number beginning to rise around P2 through P5 and then gradually decreasing at P12. Thus, ultrasonic vocalizations can be used as a milestone to evaluate early postnatal development (Hahn et al., 1998; Rieger and Dougherty, 2016) and are used as a measure of social communication in rodent models of neurodevelopmental disorders (Scattoni et al., 2009; Ey et al., 2011). Therefore, we tested the effect on the number of ultrasonic calls at P7 of developmental exposure to the MARBLES PCB mix, expression of genetic mutations that alter the fidelity of calcium signaling, singly and combined environmental and genetic factors. Developmental exposure to the MARBLES PCB mix at 0.1, 1, or 6 mg/kg in the maternal diet caused a significant decrease in the number of ultrasonic calls made by WT mice, but had no effect on the number of calls made in any of the mice expressing knock-in mutations (Figure 1A). However, genotype alone had an effect. Within each genotype, the vehicle-control group emitted significantly fewer ultrasonic vocalizations than the WT vehicle control pups (Figure 1A). There were no PCB- or genotype-induced differences in body temperature at P7 (Figure 1B). Developmental PCB exposure also had no effect on body mass. In contrast, T4826I and CGG vehicle control mice had significantly lower body mass at P7 when compared to WT vehicle control pups (Figure 1C).

### Developmental Polychlorinated Biphenyl Exposure or the T4826I Mutation Increased Repetitive Behavior

**FIGURE 2:**
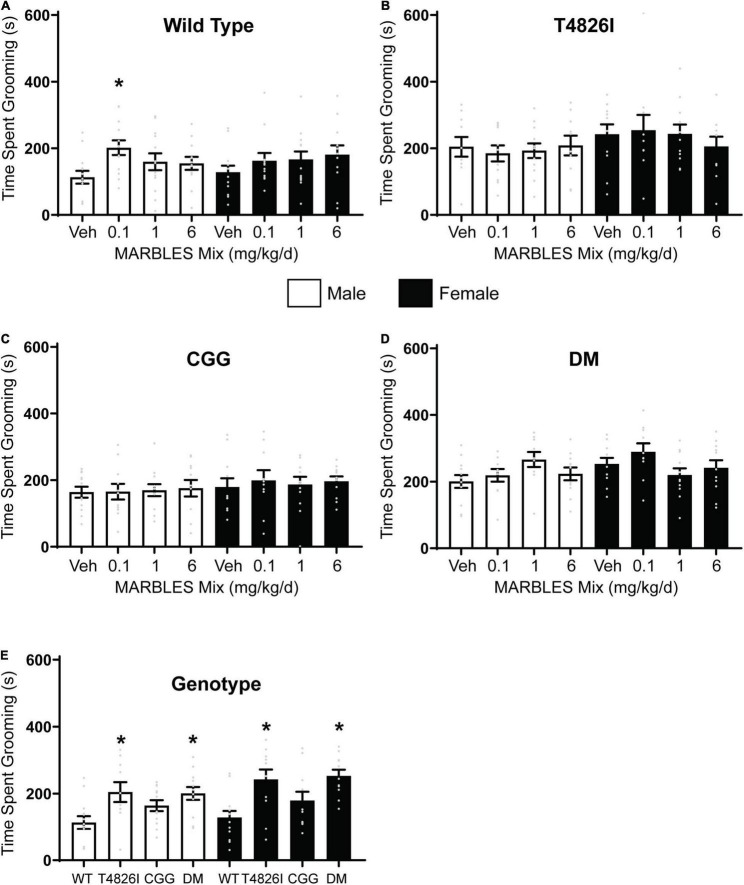
Developmental PCB exposure or expression of expression of a gain-of-function RyR mutation (T4826I) increased repetitive behavior in P26-28 mice. Repetitive behavior was assessed in one male and one female per litter per experimental group by quantifying the time spent grooming in **(A)** WT, **(B)** T4826I, **(C)** CGG, or **(D)** DM mice during a 10-min period. **(E)** Time spent grooming in vehicle control mice of each genotype. Cumulative time spent grooming is presented as mean ± SEM (*n* = 10–13). * Indicates significantly different from sex-matched **(A–D)** vehicle control or **(E)** sex-matched WT at *p* < 0.05 as determined using a one-way ANOVA or Kruskal Wallis test with *post hoc* Holm-Sidak’s multiple comparisons test or Dunn’s test, respectively.

Self-grooming is typical behavior for mice that can be leveraged as a behavioral assay for measuring abnormal repetitive or stereotypic behavior. If mice groom for a significantly longer duration than what is considered typical, it is suggestive of repetitive behavior and this has been characterized in multiple mouse models of neurodevelopmental disorders (Ey et al., 2011; Ellegood et al., 2021; Haigh et al., 2021). Developmental exposure to the lowest dose of the MARBLES mix (0.1 mg/kg/d) caused a significant increase in time spent grooming in WT male mice (Figure 2A). In contrast, developmental exposure to the MARBLES PCB mix had no effect on WT females. Exposure to PCBs had little to no effect on the grooming behavior in any of the transgenic mice when compared to their sex- and genotype-matched vehicle controls (Figures 2B–D). However, both male and female T426I and DM mice spent significantly more time grooming when compared to their WT sex-matched controls (Figure 2E).

### Developmental Polychlorinated Biphenyl Exposure and/or Genotype Alters Social Behavior

**FIGURE 3:**
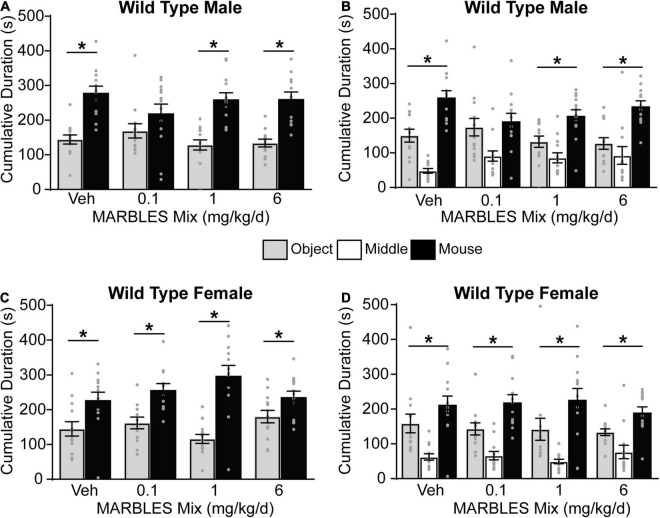
Developmental exposure to 0.1 mg/kg of MARBLES PCB mix in the maternal diet decreased sociability in male WT mice. **(A,C)** The amount of time males **(A)** or females **(C)** spent sniffing either a novel sex- and age-matched mouse vs. a novel object. **(B,D)** The amount of time males **(B)** or females **(D)** spent on each chamber side. Data presented as mean ± SEM (*n* = 12–13). * Indicates significant difference between novel mouse vs. novel object at *p* < 0.05 as determined using an unpaired *t*-test or Mann-Whitney test.

The three-chambered social approach task with automated data collection (Silverman et al., 2010; Yang et al., 2011) was used to assess changes in sociability, as deficits in this behavior are a component of multiple neurodevelopmental disorders. Vehicle control WT males and females displayed a preference for social behavior, evidenced by subjects spending significantly more time investigating a novel mouse vs. a novel object (Figures 3A,C), and by spending significantly more time in the novel mouse whole chamber side (Figures 3B,D). Developmental PCB exposure had no effect on sociability in WT females (Figures 3C,D); however, WT males in the 0.1 mg/kg/d MARBLES PCB mix dose group exhibited significantly reduced sociability (Figures 3A,B).

**FIGURE 4:**
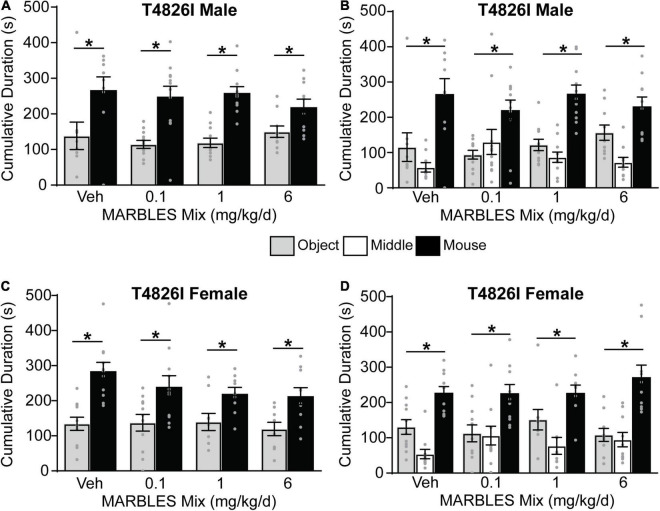
Developmental exposure to the MARBLES PCB mix did not affect social behavior in T4826I mice. **(A,C)** The amount of time males **(A)** or females **(C)** spent sniffing either a novel sex- and age-matched mouse or a novel object. **(B,D)** The amount of time males **(B)** or females **(D)** spent on each chamber side. Data presented as mean ± SEM (*n* = 9–12). * Indicates significant difference between novel mouse vs. novel object at *p* < 0.05 as determined using an unpaired *t*-test, Mann-Whitney test or unpaired *t*-test with Welch’s correction.

**FIGURE 5:**
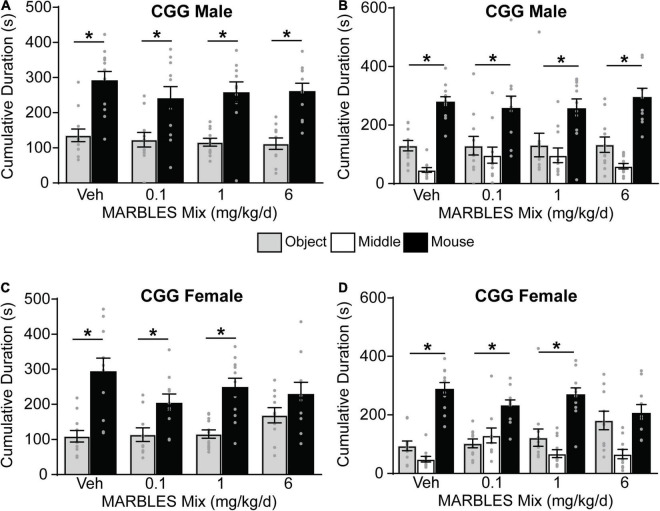
Developmental exposure to MARBLES PCB mix at 6 mg/kg in the maternal diet decreased sociability in female CGG mice. **(A,C)** The amount of time males **(A)** or females **(C)** spent sniffing either a novel sex and age matched mouse or a novel object. **(B,D)** The amount of time males **(B)** or females **(D)** spent on each chamber side. Data presented as mean ± SEM (*n* = 10–12). * Indicates significant difference between novel mouse vs. novel object at *p* < 0.05 as determined using an unpaired *t*-test, Mann-Whitney test or unpaired *t*-test with Welch’s correction.

**FIGURE 6:**
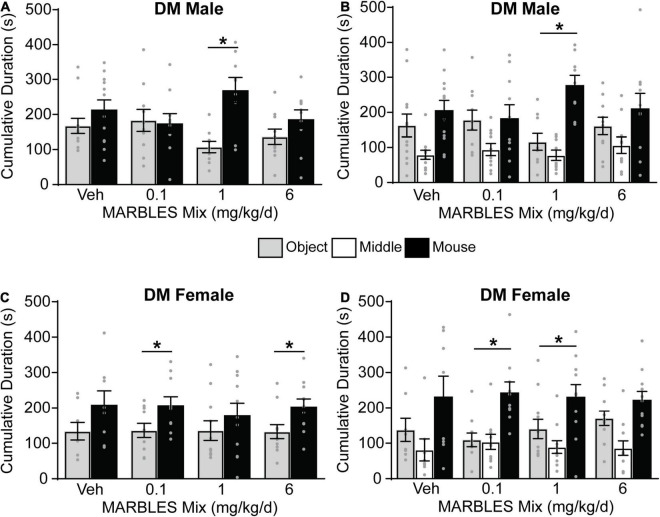
Sociability in male and female DM mice was altered by genotype and PCB exposure. **(A,C)** The amount of time males **(A)** or females **(C)** mouse spent sniffing either a novel sex- and age-matched mouse or a novel object. **(B,D)** The amount time males **(B)** or females **(D)** spent in each chamber side. Data presented as mean ± SEM (*n* = 8–12). * Indicates significant difference between novel mouse vs. novel object at *p* < 0.05 as determined using an unpaired *t*-test, Mann-Whitney test or unpaired *t*-test with Welch’s correction.

Vehicle control T4826I mice showed a preference for social behavior, with developmental exposure to the MARBLES mix having no effect on the sociability of either T4826I male and female mice (Figure 4). Vehicle control CGG mice also displayed a preference for social behavior. The sociability of CGG males was not affected by the MARBLES mix (Figures 5A,B). In contrast, CGG females in the 6 mg/kg/d MARBLES PCB dose group displayed reduced sociability (Figures 5C,D). Both male and female vehicle control DM mice displayed reduced social behavior (Figure 6). Developmental exposure to the MARBLES mix altered this phenotype, with females in the 0.1 and males in the 1 mg/kg dose groups exhibiting increased sociability (Figures 6A–D). There was also suggestive evidence that the 1 or 6 mg/kg doses increased sociability in DM females, but this varied depending on the parameter measured, e.g., close-proximity sniffing measure (Figure 6C) or time spent in the chamber side (Figure 6D). Changes in sociability were not due to differences in exploration, as there were no differences in total side entries for any genotype (Supplementary Figure 2), a key motor control, which is often omitted from reports, leading to misinterpreted data (Silverman et al., 2020; Ellegood et al., 2021). Additionally, there were no differences in velocity amongst the groups (Supplementary Figure 3) indicating that the changes in behavior are unlikely due to changes in overall activity.

Differences in body mass or brain development could potentially contribute to differences in behavior. Changes in body mass observed at P7 are still apparent at P27-32, with both male and female T4826I and CGG mice having significantly less body mass than their sex -matched WT controls (Supplementary Figure 4). Similarly, DM male mice weighed less than male WT mice. T4826I and DM mice had significantly less brain mass than WT controls, but when brain mass was normalized to body weight, only T4826I males and CGG mice exhibited a significant difference in brain mass relative to body mass (Supplementary Figure 4). PCB exposure had no effect on body mass, brain mass or the ratio of brain to body mass in WT or DM pups (Supplementary Figure 5). However, PCB exposure significantly increased body mass and decreased the brain to body mass ratio in female T4826I females in the 1 and 6 mg/kg dose groups, and significant decreased the brain to body mass in CGG males in the 0.1 and 1 mg/kg dose groups (Supplementary Figure 5).

### Developmental Polychlorinated Biphenyl Exposure Had Minimal Effect on T3 and T4 Levels

**FIGURE 7:**
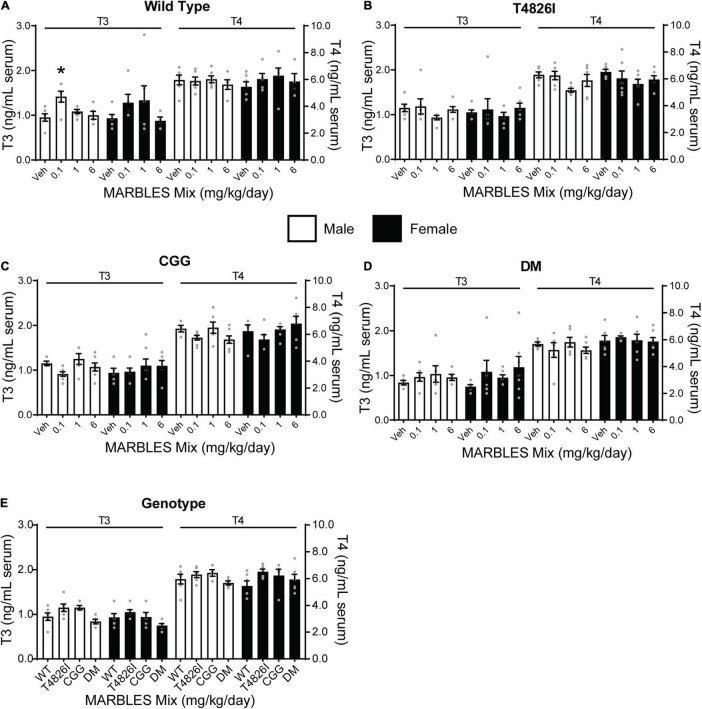
Developmental PCB exposure and genotype had had minimal to no effect on thyroid hormone levels. **(A–D)** The serum levels of triiodothyronine (T3) and thyroxine (T4) in **(A)** WT, **(B)** T4826I, **(C)** CGG, and **(D)** DM mice developmentally exposed to varying doses of the MARBLES PCB mix in the maternal diet. **(E)** T3 and T4 levels in vehicle control mice of each genotype. Data presented as mean ± SEM (*n* = 4–7). * Indicates significant difference between sex-matched vehicle control at *p* < 0.05 as determined using a one-way ANOVA or Kruskal Wallis test with *post hoc* Holm-Sidak’s multiple comparisons test or Dunn’s test, respectively.

Thyroid hormone (TH) is a critical factor for normal neurodevelopment (Oppenheimer and Schwartz, 1997; Williams, 2008), and exposure to PCBs has been shown to decrease circulating T3 and/or T4 levels (Oppenheimer and Schwartz, 1997; Kato et al., 2003; Williams, 2008; Martin and Klaassen, 2010). Therefore, we assessed whether developmental exposure to the MARBLES PCB mix altered TH levels in juvenile mice. There were no significant effects of PCB exposure on T3 or T4 levels within each genotype and sex, with the exception of increased T3 levels in the 0.1 mg/kg WT males (Figures 7A–D). There were no significant effects of genotype on T3 or T4 levels relative to sex-matched WT vehicle controls (Figure 7E).

### Total Polychlorinated Biphenyl Levels in Pup Brains Is Dose-Dependent and Not Affected by Genotype or Sex

**FIGURE 8:**
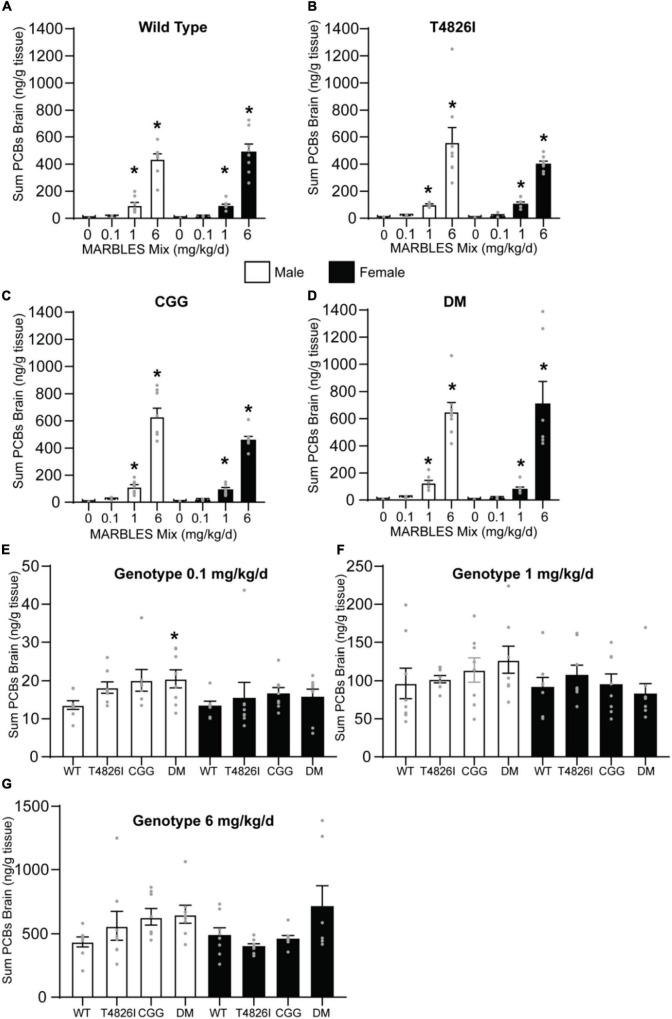
PCB brain burden increased in a dose-dependent manner independent of genotype. **(A–D)** The sum PCB levels in brain tissue of **(A)** WT, **(B)** T4826I, **(C)** CGG, or **(D)** DM mice developmentally exposed to varying doses of the MARBLES PCB mix in the maternal diet. **(E)** The sum PCB levels in the brain tissue of each genotype at the 0.1, **(E)** 1, **(F),** or 6 **(G)** mg/kg/d exposure group. Data presented as mean ± SEM (*n* = 7–9). The sum of PCBs that were above 70% detection frequency in the 1 and 6 mg/kg/d groups were used and include PCB 28, 52, 101, 118, 138, 153, and 180. * Indicates significant difference from sex-matched vehicle control at *p < 0.05 as determined using a one-way ANOVA or Kruskal Wallis test with *post hoc* Holm-Sidak’s multiple comparisons test or Dunn’s test, respectively. Detailed information on individual PCB congener content can be found in Excel spreadsheets in Supplementary Data Sheet 2.

Differences between groups in the total burden of PCBs in the brain could contribute to some of the group differences in behavior observed in this study. To address this possibility, we measured the PCB levels in P27-32 brain tissue. Only PCB congeners with a detection frequency greater than 70% in the 1 and 6 mg/kg dose groups were used to create a sum PCB value that was then used to assess PCB levels across groups. There is a clear dose response in each genotype, with the PCB brain burden significantly increasing as the MARBLES PCB dose increased (Figures 8A–D). There was no effect of genotype on PCB brain burden in the 1 or 6 mg/kg dose groups (Figures 8F,G); however, there was a significant increase in brain PCB levels in the 0.1 mg/kg DM males compared to 0.1 mg/kg WT males (Figure 8E). Detailed information on individual PCB congener content can be found in Excel spreadsheets in Supplementary Material.

## Discussion

It is widely posited that individual risk for many neurodevelopmental disorders is determined by complex interactions between genetic and environmental risk factors (Lyall et al., 2017; Bolte et al., 2019; Cheroni et al., 2020); however, the identification of specific gene-environment interactions that influence individual risk for neurodevelopmental disorders remains a significant data gap (Masini et al., 2020). To address this data gap, we leveraged the MARBLES PCB mix, the composition of which is based on the PCB congener profile detected in the serum of pregnant women at increased risk of having a child with a neurodevelopmental disorder (Sethi et al., 2019), and three lines of mice genetically altered to express human-relevant mutations that alter calcium signaling (Barrientos et al., 2012; Robin et al., 2017; Keil et al., 2019b) to test two hypotheses: (1) developmental exposure to the human-relevant MARBLES PCB mixture causes behavioral phenotypes relevant to neurodevelopmental disorders; and (2) expression of heritable mutations that alter Ca^2+^ signals modulate the behavioral effects of developmental PCB exposure. The data reported in this study support both hypotheses and add to the evidence implicating PCBs, and in particular RyR-active PCBs (Granillo et al., 2019), as environmental risk factors for neurodevelopmental disorders (Panesar et al., 2020).

The most direct evidence in support of the hypothesis that developmental PCB exposure results in behavioral phenotypes relevant to neurodevelopmental disorders is our observations of decreased ultrasonic vocalizations at P7, increased self-grooming at P25-P30, and lack of typical three-chambered sociability at P27-P32 in WT mice exposed to the MARBLES PCB mix in the maternal diet throughout gestation and lactation. While significant PCB effects on ultrasonic vocalizations were evident in WT mice in all PCB dose groups, significant PCB effects on grooming and social approach in WT mice were observed only in males in the 0.1 mg/kg PCB dose group. The mechanism(s) underlying this non-monotonic dose-response relationship are not known, but are consistent with previous reports of non-monotonic dose-related effects of developmental exposure to Aroclor 1254 (Yang et al., 2009) and PCB 95 (Wayman et al., 2012b), a RyR-active non-dioxin-like congener (Pessah et al., 2010), on dendritic arborization as well as spatial learning and memory in rats. The observation that deficits in social reciprocity and repetitive behavior were observed in PCB-exposed WT males but not WT females mimics the sex bias of autism spectrum disorder, which affects 4 times as many boys as girls, on average. This observation suggests that sex-specific responses to environmental factors may contribute to sex biases in the prevalence and/or severity of neurodevelopmental disorders. The relevance of these findings to the human condition is supported by several observations. First, these behavioral assays have face validity to core clinical signs of autism spectrum disorder; specifically, repetitive behavior and deficits in social reciprocity and communication (Silverman et al., 2010; Ey et al., 2011). Second, the PCB mixture to which mice were exposed reflects the PCB congener profile that actually exists in the gestational environment of at-risk individuals (Sethi et al., 2019). Third, the PCB brain burden in pups exposed to the MARBLES PCB mix at 0.1 mg/kg/d in the maternal diet, which ranged from 6.2 to 43.6 ng/g wet weight, is comparable to the range of PCB levels reported in human brain tissue (6–66 ng/g wet weight) (Chu et al., 2003; Hatcher-Martin et al., 2012; Klocke et al., 2020).

To the best of our knowledge, ultrasonic vocalizations in young pups have been investigated in only one other published study of PCB developmental neurotoxicity. Intraperitoneal injection of Aroclor 1221 into rat dams at 1 mg/kg on P16, P18, and P20 had no effect on affiliative ultrasonic vocalizations of offspring tested at P1-P3 (Bell et al., 2016). The discrepant finding between this study and ours likely reflects significant differences in study design (species, PCB dosing paradigm, ultrasonic vocalization testing protocol). In contrast, our observations that developmental PCB exposure increases grooming and decreases sociability in young male, but not female, WT mouse pups are largely consistent with previously published observations. For example, oral administration of PCB 126, a dioxin-like congener, to rat dams at 30 μg/kg on gestational day 15, significantly increased the time male offspring spent grooming (Orito et al., 2007). This same study (Orito et al., 2007), as well as another study of rats exposed developmentally to an equal mixture of PCB 47 and PCB 77, non-dioxin-like and dioxin-like congeners, respectively, at 12.5 or 25 mg/kg in maternal chow throughout gestation and lactation (Jolous-Jamshidi et al., 2010), reported decreased sociability in juvenile males. A study of the sociability of CD1 mice exposed to a mixture of six PCBs (28, 52, 101, 138, 153, and 180) in maternal chow at 10 or 1,000 ng/kg throughout gestation and lactation revealed PCB effects on not only males, evidenced as increased sociability at P50, but decreased sociability at P330, but also females, reported as increased sociability at P330. Given the significant differences in study design between this study and ours (mouse strain, PCB exposure and age at the time of testing), it is difficult to make direct comparisons; however, the collective data across all studies suggest that developmental PCB exposure alters social behavior, but the effect varies according to sex, species/strain, age of testing, and/or PCB congener/dose.

Consistent with the observation that species and/or strain influence the expression of behavioral phenotypes following developmental PCB exposures, we observed that genotype altered the behavioral effects of the MARBLES PCB mix. Specifically, in contrast to observations of WT mice, ultrasonic vocalizations were not significantly different in any PCB dose group in any of the three transgenic lines expressing heritable human mutations that modulate calcium signaling relative to genotype-matched vehicle controls. Similarly, PCB exposure had no effect on repetitive behavior in T4826I, CGG, or DM mice of either sex. However, PCB exposure decreased sociability in CGG females in the 6 mg/kg dose group and increased sociability in DM males in the 1 mg/kg group and DM females in the 0.1, 1 and 6 mg/kg groups. The differences in behavioral responses to the MARBLES PCB mix were likely not due to genotype-specific differences in PCB levels in the brain tissue of pups. While brain levels of PCBs varied in a dose-dependent manner, within any dose group, levels did not differ between genotypes with one exception: 0.1 mg/kg DM males had a higher PCB brain burden than 0.1 mg/kg WT males. Collectively, these data suggest that expression of a heritable mutation in Ca^2+^ signaling partially mitigated the impacts of developmental exposure to the MARBLES PCB mix on social behavior and repetitive behavior; whereas expression of more than one of these mutations altered PCB effects, both in terms of sex dependency and direction of effect.

Interestingly, expression of one or more heritable mutations that alter the fidelity of neuronal Ca^2+^ signals altered behavioral outcomes independent of developmental PCB exposures, and the outcome largely phenocopied PCB-induced behavioral phenotypes (Table 1). In all three genotypes, ultrasonic vocalizations were significantly decreased relative to WT vehicle control. In contrast, increased grooming was observed in T4826I and DM mice, but not CGG mice, suggesting that the gain-of-function *RYR1* mutation plays a predominant role in driving this behavioral phenotype. Decreased sociability was observed only in DM mice, consistent with our previous characterization of sociability in these same transgenic lines (Keil et al., 2019b), suggesting that gene dosage is an important determinant of this phenotype. The observation that increased repetitive behavior and deficits in sociability can be independently affected suggests that different molecular mechanisms contribute to each behavioral deficit. Interestingly, in contrast to PCB effects on WT mice, which were male-selective, the effect of genotype on grooming and sociability was observed in both sexes. The biological explanation for these genotype-dependent differences in sex bias remains to be determined.

**TABLE 1:** Summary of PCB and genotype effects on behavior compared to wildtype (WT) vehicle control mice.

	WT males (0.1 mg/kg/d)	T4826I* (vehicle)	CGG* (vehicle)	Double mutant* (vehicle)
USVs (communication)	Decrease	Decrease	Decrease	Decrease
Grooming (repetitive behavior)	Increase	Increase	No change	Increase
Social behavior	Decrease	Decrease	No change	Decrease

*Both sexes of the transgenic mice showed the same phenotype listed in this table.

Our data suggest that environmentally relevant PCB exposures during brain development modulate molecular mechanisms targeted by the gain-of-function *RYR1* and CGG mutations to alter behavior. While diverse mechanisms have been proposed to mediate PCB developmental neurotoxicity, two of the more prevalent hypotheses include disruption of thyroid hormone homeostasis and altered fidelity of Ca^2+^ signaling (reviewed in Pessah et al., 2019; Klocke and Lein, 2020). Several lines of evidence from the current study provide weak support for the former, but strong support for the latter. First, we previously demonstrated that the PCB congeners that comprise the MARBLES PCB mixture neither agonize nor antagonize the thyroid hormone receptor singly or in combination; in contrast, the MARBLES PCB mixture has potent RyR activity as determined by equilibrium binding of [^3^H]ryanodine to RyR1-enriched microsomes (Sethi et al., 2019). Second, developmental exposure to the MARBLES mixture in the maternal diet throughout gestation and lactation did not significantly alter serum T3 or T4 levels in WT or transgenic mice, with the exception of increased T3 in the 0.1 mg/kg WT males. Since this later group exhibited PCB effects on ultrasonic vocalizations, grooming and social behavior, we cannot rule out the possibility that increased T3 contributed to these behavioral phenotypes. However, while a few studies have shown changes in behavior linked to decreased T3 levels (Roman et al., 2013; Fetene et al., 2017), there are no data of which we are aware that demonstrate a link between excessive serum T3 levels and behavioral outcomes relevant to neurodevelopmental disorders. Moreover, there were no differences in T3 levels between vehicle control WT and DM mice despite striking genotype-specific differences in grooming behavior and sociability. Third, there was significant overlap in the behavioral phenotypes exhibited by WT mice developmentally exposed to PCBs and mice that expressed heritable mutations in Ca^2+^ signaling, particularly expression of the T4826I mutation in *RYR1*, singly or in combination with the CGG mutation. These observations suggest shared molecular mechanisms of Ca^2+^ dysregulation. In support of this hypothesis, multiple groups have shown that genetic alterations that dysregulate Ca^2+^ signaling caused deficits in social behaviors (Matsuo et al., 2009; Bader et al., 2011; Nakao et al., 2015; Kabir et al., 2017). In one of these studies, mice engineered to express mutant gain-of-function calcium channels also exhibited increased repetitive behaviors (Bader et al., 2011). In a separate study, reduced calcium signaling in astrocytes was also associated with increased grooming behavior in mice (Yu et al., 2018). An outstanding question is why expression of heritable mutations that increase Ca^2+^ signaling did not increase sensitivity to the developmental neurotoxicity of PCB congeners that similarly increase Ca^2+^ signaling, evident as a leftward shift in the dose-response curve. One potential explanation is that the genetically determined phenotypes are an all-or-none phenomenon, although the observation that DM mice, but not T4861I or CGG mice, exhibit decreased sociability argues against this possibility.

## Conclusion

In conclusion, our studies demonstrated that developmental exposure to a human-relevant PCB mixture phenocopied social behavior phenotypes observed in mice expressing heritable human mutations that increase Ca^2+^ signaling, and that expression of these mutations altered the effects of developmental PCB exposure on sociability. One key difference between the PCB exposure vs. genotype effects was the sex-specificity of the outcome: PCB effects on repetitive behavior and sociability were male-specific, whereas genotype effects on these behaviors was sex-independent. These findings suggest that: (1) developmental PCB exposures can cause behavioral phenotypes of relevance to neurodevelopmental disorders, but this effect varies according to sex and genotype; and (2) sex-specific responses to environmental factors may contribute to sex biases in the prevalence and/or severity of neurodevelopmental disorders.

## Data Availability Statement

The original contributions presented in the study are included in the article/Supplementary Material, further inquiries can be directed to the corresponding author/s.

## Ethics Statement

The animal study was reviewed and approved by University of California, Davis Institutional Animal Care and Use Committee.

## Author Contributions

IP and PL conceptualized the project and obtained funding to support the work. PL supervised all aspects of this study. JS oversaw the behavioral studies. BP oversaw the PCB analyses. SS, KK, JS, and PL designed the experiments. SS and KK maintained the mouse colony, dosed the animals, conducted the behavioral studies, collected tissues for PCB quantitation, and analyzed the behavioral data. SS, AV, and BP optimized the protocol for PCB quantitation. SS and AV conducted the PCB analyses. AV analyzed the PCB analyses data. CK facilitated the analysis of tissue samples for thyroid hormone levels. SS, KK, and CK composed the figures. SS wrote the initial manuscript draft. KK and PL made significant edits to the early versions of the manuscript. All authors reviewed and made final edits to the manuscript prior to submission.

## Conflict of Interest

The authors declare that the research was conducted in the absence of any commercial or financial relationships that could be construed as a potential conflict of interest.

## Publisher’s Note

All claims expressed in this article are solely those of the authors and do not necessarily represent those of their affiliated organizations, or those of the publisher, the editors and the reviewers. Any product that may be evaluated in this article, or claim that may be made by its manufacturer, is not guaranteed or endorsed by the publisher.
