# Supplementary material for: Developmental Exposure to a Human-Relevant Polychlorinated Biphenyl Mixture Causes Behavioral Phenotypes That Vary by Sex and Genotype in Juvenile Mice Expressing Human Mutations That Modulate Neuronal Calcium
Source: Front Neurosci. 2021 Dec 6;15:766826. doi: 10.3389/fnins.2021.766826 (PMC8685320; doi:10.3389/fnins.2021.766826)
Supplement: Supplementary file 1 [file Data_Sheet_1.docx]

Supplementary Material

# Supplementary Figures


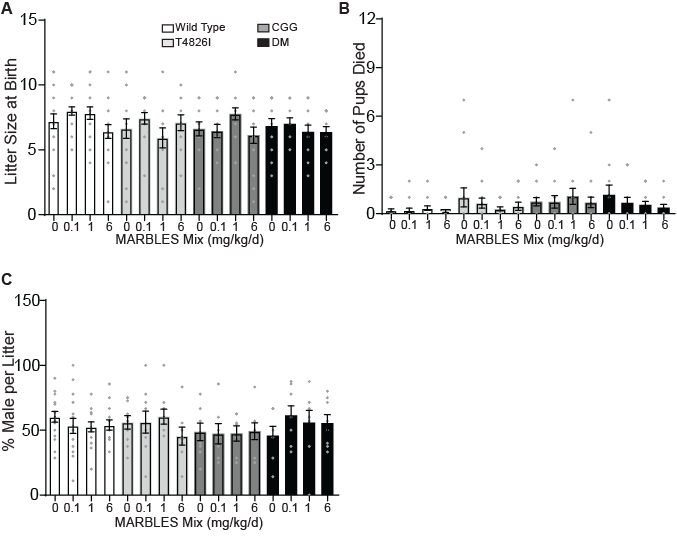


**Supplemental Figure S1. Developmental PCB exposure or expression of mutations that alter calcium signaling do not alter litter size or sex ratio.** **(A)** The total number of pups per litter, n=10-20 litters per group. **(B)** The number of pups that died after birth, n=11-20 litters per group. **(C)** The percentage of male pups per litter, n=7-17 litters per group. Dams were checked daily, litter size was determined from live pups found the first morning after parturition, number of dead pups was determined by those found in the cage or when number at weaning was less than the number at birth. Sex ratio was determined from litters for which sex was determined for all pups. Results are mean ± SEM. No significant differences were found as determined using one-way ANOVA or Kruskal-Wallis test (p<0.05).


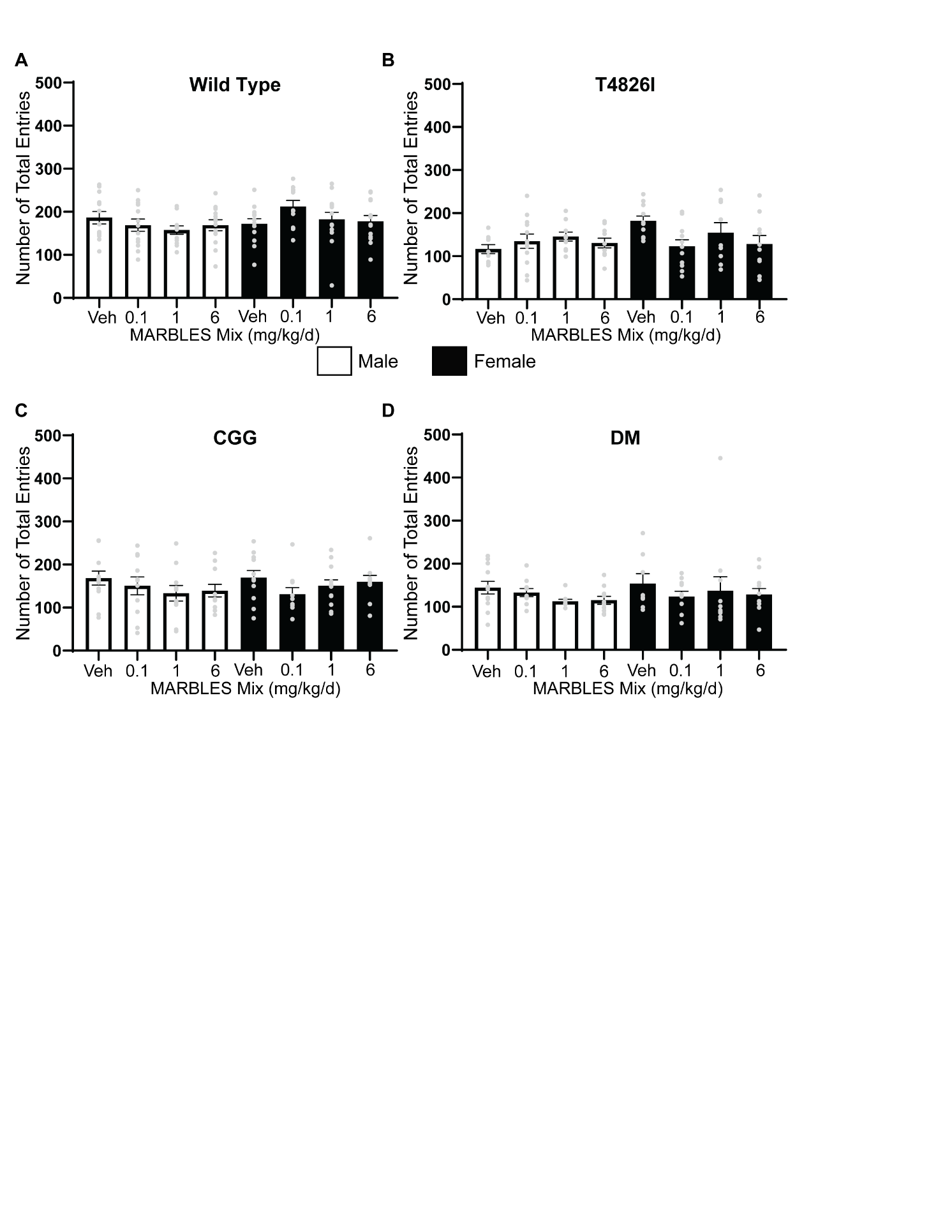


**Supplemental Figure S2. Developmental exposure to the MARBLES PCB mix did not influence the total number of chamber side entries during social approach testing.** The number of total entries of **(A)** wild type, **(B)** T4826I, **(C)** CGG, **(D)** DM male or female mice during social approach. Data presented as mean ± SEM (n = 8-13) with dots representing individual subjects. There were no significant differences between groups at p < 0.05 as determined using one-way ANOVA, Welch’s ANOVA or Kruskal Wallis test.


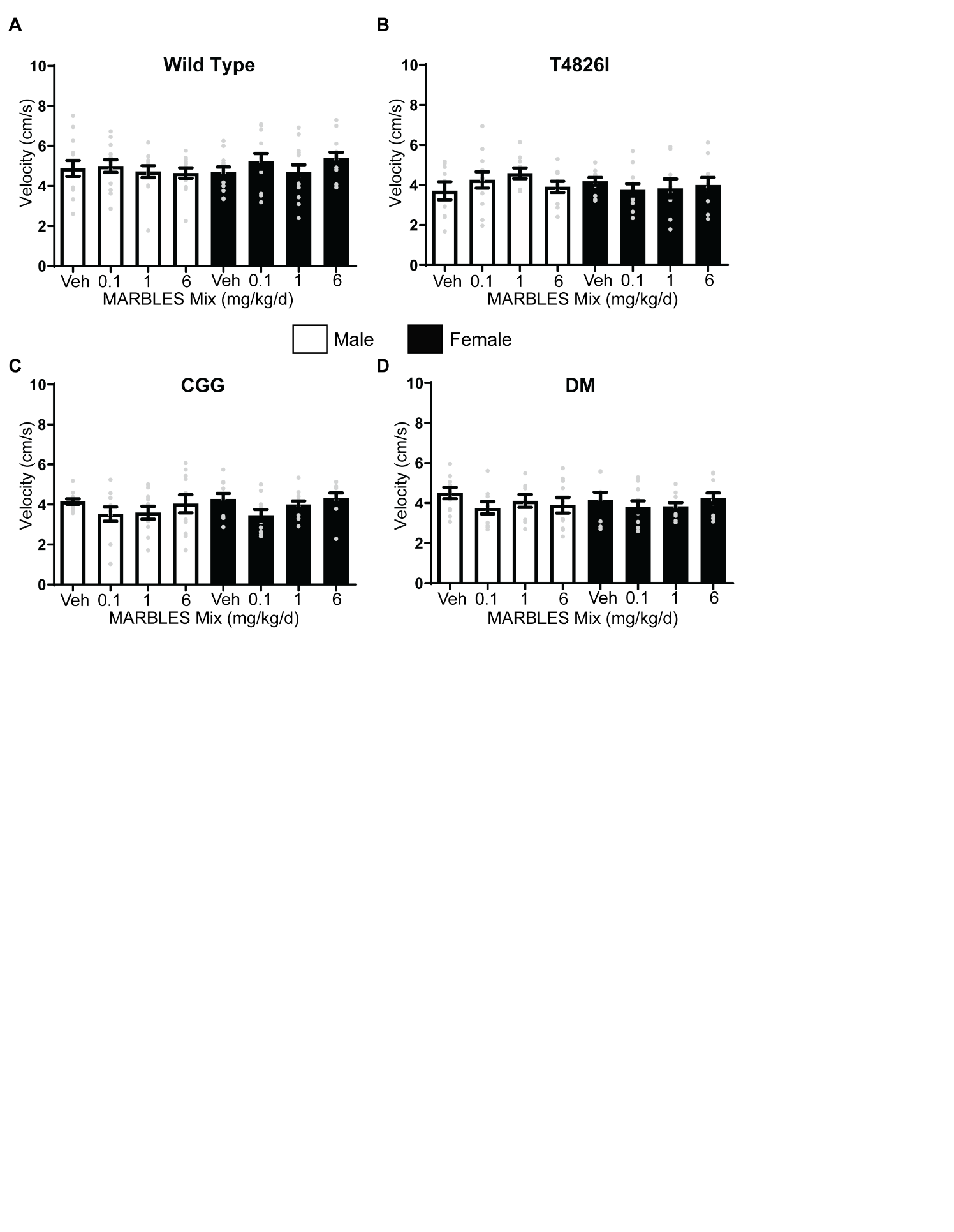


**Supplemental Figure S3.** **Developmental exposure to the MARBLES PCB mix did not affect velocity during social approach testing.** The velocity of **(A)** wild type, **(B)** T4826I, **(C)** CGG, **(D)** DM male or female mice during social approach. Data presented as mean ± SEM (n = 8-13) with dots representing individual subjects. No significant differences were found at p < 0.05 as determined using one-way ANOVA or Kruskal Wallis test.


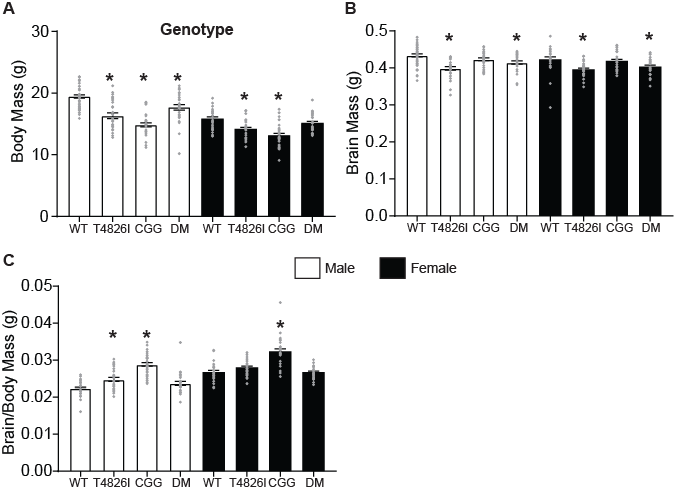


**Supplemental Figure S4. Expression of mutations that alter calcium signaling alter brain and body mass.** **(A)** Body mass of vehicle control animals in each genotype at time of tissue collection PND 27-32. **(B)** Brain mass of vehicle control animals in each genotype at time of tissue collection PND 27-32. **(C)** Normalized brain to body mass ratios of vehicle control animals in each genotype. Data are presented as the mean ± SEM (n=27-44). *Significantly different from same sex WT at p < 0.05 as determined using one-way ANOVA or Kruskal Wallis test with Holm-Sidak’s multiple comparisons test or Dunn’s test respectively. Data for males represented by the open bars; data for females represented by black bars.

**Supplemental Figure S5. Developmental PCB exposure and expression of mutations that alter calcium signaling alter brain to body mass ratio**. **(A-D)** Body mass at time of tissue collection PND 27-32 of **(A)** WT, **(B)** T4826I, **(C)** CGG, or **(D)** DM mice developmentally exposed to varying doses of the MARBLES PCB mix in the maternal diet. **(E-H) Brain mass** at time of tissue collection PND 27-32 of **(E)** WT, **(F)** T4826I, **(G)** CGG, or **(H)** DM mice developmentally exposed to varying doses of the MARBLES PCB mix in the maternal diet. **(I-L)** Normalized brain to body mass ratios at time of tissue collection PND 27-32 of **(I)** WT, **(J)** T4826I, **(K)** CGG, or **(L)** DM mice developmentally exposed to varying doses of the MARBLES PCB mix in the maternal diet. Data are presented as the mean ± SEM (n=23-47). *Significantly different from sex-matched WT at p < 0.05 as determined using one-way ANOVA, one-way ANOVA with Welch’s correction or Kruskal Wallis test with Holm-Sidak’s multiple comparisons test, Dunnett’s T3 multiple comparisons test or Dunn’s test respectively. Data for males represented by the open bars; data for females represented by black bars.
